# Chick Begging Calls Reflect Degree of Hunger in Three Auk Species (Charadriiformes: Alcidae)

**DOI:** 10.1371/journal.pone.0140151

**Published:** 2015-11-04

**Authors:** Anna V. Klenova

**Affiliations:** Department of Vertebrate Zoology, Faculty of Biology, Lomonosov Moscow State University, Vorobievy Gory, 1/12, Moscow, 119234, Russia; Universite Paris Sud, FRANCE

## Abstract

Begging behaviour is an important element in the parent-offspring conflict; it has been studied in many avian species. However, the majority of the studies have been entirely based on the call counts, and they agreed that vocal activity was a good indicator of chick’s nutritional need and/or condition. Fewer researches were dedicated to the temporal-frequency variables of the begging calls themselves and they showed contrary results. Here begging behaviour in three burrow nested, uniparous species of auks (Alcidae) was studied. These objects provide an opportunity to study the signalling value of begging calls in the absence of important confounding factors such as nestling competition and predation pressure. I recorded calls of individual chicks in two conditions: during natural feeding and after experimental four-hour food deprivation. I found that almost all measured acoustic variables contain information about the chick’s state in all studied species. The hungry chicks produced calls higher in fundamental frequency and power variables and at higher calling rate compared to naturally feeding chicks. The effect of food deprivation on most acoustic variables exceeded both the effects of individuality and species. In all studied species, the frequency variables were stronger affected by hunger than the calling rate and call durations. I suppose that such strong change of acoustic variables after food deprivation can be explained by absence of vocal individual identification in these birds. As parents do not need to check individuality of the chick in the burrow, which they find visually during the day time, the chicks could use all of the acoustic variables to communicate about their nutritional needs.

## Introduction

In many bird species, nestlings use various signals for food begging [1, 2]. It is well known that chicks communicate their needs by begging, and parents use this information to adjust their investment [3]. Cues to offspring fitness and demand could be expressed in chick displays, like mouth colour, gaping, postures and behaviour, and influence parental provisioning [1, 4–6].

Vocalization is the most common form of begging behaviour in birds. Vocal begging displays have been well studied especially in passerines, and provide a good model for investigating the evolution of animal signals [1, 7, 8]. The narrow taxonomic scope of research on this topic limits our understanding; for example, do universal acoustic indicators of chick hunger or fitness exist? Almost all studies have shown that begging vocal activity (usually quantified as the number or rate of begging calls within the begging session) are correlated with nutritional needs [2, 4 8–13]. Fewer studies have investigated acoustic structure (e.g. fundamental frequency modulation, frequency spectrum) and they have produced variable results. For example, changes in frequency parallel degree of hunger in some species [13–15], but not others [11, 16–19].

I studied acoustic properties of chick begging calls in relation to hunger level in three species of auk (Charadriiformes: Alcidae). Many seabirds, like Alcidae or Procellariiformes, are ideal objects for such research because most species have an obligate clutch size of one egg, so begging behavior is not complicated by sibling competition. Also, many seabirds nest in deep burrows so parents can not assess the chick condition visually, and must rely on cues from vocal begging [20, 21]. Vocal begging has been studied in detail in many Procellariiformes [15–19, 22–25], but not in auks (only two of 24 extant species has been studied) [9; 26].

The species in this study were crested auklet (*Aethia cristatella*), parakeet auklet (*Cyclorrhynchus psittacula*), and horned puffin (*Fratercula corniculata*). All species have semi-precocial chick development and nest in burrows; parents feed their single chick only in the nest, and the relationship between adults and juveniles apparently ceases immediately at fledging [20]. The duration of chick growth and development is highly variable, reflecting intermittent provisioning by parents, and varies from 27–36 days in duration for crested and parakeet auklets [27, 28] to 34–43 days for horned puffin [29]. Previous studies of crested auklet and horned puffin described chick vocal repertoires and vocal development from hatching to fledging [30]. Those studies also revealed low individuality in chick calls, which can be related to their solitary lives in burrows [31]. In this study, I compare the frequency and temporal variables of chick begging calls emitted during natural food provisioning and after experimental food deprivation.

## Materials and Methods

### Ethics Statement

No specific permissions were required for our study location or species according to sections §44 and §6 of the Federal Law of the Russian Federation No. 52 from 24.04.1995 (last update 07.05.2013) “On Wildlife”. There were no Special Protected Natural Territories in my study area (described below in section “Study area and nest-burrow inspection”, including coordinates), and my study species are not listed in the Red List of the Russian Federation. My activities (described below in sections “Study area and nest-burrow inspection” and “Sound recordings”) did not include withdrawal of investigated species from nature. Data collection protocol #2013–48 was approved by the Committee of Bio-ethics of Lomonosov Moscow State University.

### Study area and nest-burrow inspection

The study was conducted on Talan Island, in the northern Sea of Okhotsk, North Pacific (59°18' N, 149°05' E). The area of the island is approximately 2.5 km^2^. It contains one of the largest seabird colonies in northeastern Asia (~ 1.4 million individuals of 11 species). Populations of crested auklets, parakeet auklets and horned puffins are estimated at 260,000–300,000, 3,000–4,000 and 100,000–105,000 individuals, respectively [32]. The study was carried out during the middle part of the breeding season: 25 June to 15 August 2013.

The study was done in four study plots, each about 300–350 m^2^, located on talus and vegetated slopes of the island. Most burrows of the object species are situated deep in rock crevices or in turf and are not accessible to researchers, so I could use only ~ 10–15% all nests (nests with straight entrances in which the chick or incubating bird were visible from the surface). In the beginning of the work (25 June– 1 July 2013) I made one overall survey of each study plot and marked all usable burrows with small flags on the colony surface. During the period of hatching, I examined these nests every two days to determine the precise (± 1 day) day of hatching of each chick.

The dates of hatching in 2013 were: crested auklet, 29 June to 5 July; parakeet auklet, 15 to 28 July; and horned puffin, 23 July to 3 August [33]. To minimize disturbance, I examined nest sites only between morning and evening peaks of adult colony attendance (from 14:00 to 17:00 hours); during this time only incubating adults usually stay in the nest burrows, whereas the other parent and nonbreeding birds feed at sea [27–29].

### Sound recordings

I stopped making regular nest inspections after hatching, to prevent disturbance to provisioning parents. Each studied chick was recorded twice. The first recording session (control recording) of chick begging calls was done at 12–15 days of age, when chicks of these species are able to maintain their own body temperature and spend most of their time alone in the burrow [27–29]. I made the control recordings during the morning peak of food delivery (6:00–11:00 hours). Between 5:30–5:45 I placed the recorder (details below) in the focal burrow, programmed the recorder and immediately left the area. The recorders were programmed to run for 5 hours; I removed the equipment at 14:00 hours (by this time of day provisioning had nearly ceased). Five identical sets of recorders allowed me to collect calls from up to five chicks simultaneously.

The day after the control recording, in the evening (four hours before sunset, 19:30), I covered the burrow entrance with a net to prevent entry of provisioning parents. Next morning at 5:00–5:30, I removed the net and conducted a second recording session (experimental recording) per the protocol described above. Parents of the study species feed chicks 1–6 times during the day, with peaks in the morning and evening [27–29, 33]. Therefore experimental chicks missed at least one feeding event in the evening, and should have been hungrier the next morning than in control recording sessions. In total, I recorded 7 crested auklet, 9 parakeet auklet and 8 horned puffin chicks. Recordings were conducted on the following dates: crested auklet, 17–20 July; parakeet auklet, 28–31 July; and horned puffin, 10–13 August. During these periods weather conditions were normal (~sunny, with no strong wind and rain), and adult birds did not have obvious problems provisioning their chicks. After the experiments I inspected all the nests for 3–7 more days and found all chicks alive and developing normally. Used data collection protocol did not take into account that food deprivated chicks were both hungrier and older by one day. However, I supposed that one day of life could not change the acoustic variables of chick calls significantly, since previous study of vocal development in these species showed that all call parameters remain particularly unchanged during first 30–40 days of life [30].

For recordings I used five Sony ICD-SX712 digital recorders (sampling frequency 48 kHz) with built-in microphones. The recorders were fixed with a sticky tape on a side wall of the burrow at 0.3–0.5 m from the entrance. Burrows were 0.5–2 m in depth, so the distance between focal chick and recorder was ~0.5–1.5 m. Chicks rarely called in the absence of parents, but displayed explosive vocal activity when one of the parents entered the burrow. I registered from one to four food delivery events during the recording sessions.

### Sound and statistical analyses

I analyzed calls with Avisoft SAS-Lab Pro v. 5.1.23 (Avisoft Bioacoustics, Berlin, Germany). To increase frequency resolution of sound presentation in the spectrogram window of the program, chick calls were downsampled to 22.05 kHz with antialiasing filtering. Spectrograms were created with settings 1024-point FFT, Hamming window, frame 25% and overlap 98.43%, providing time resolution of 0.73 ms and frequency resolution of 22 Hz.

**Fig 1:**
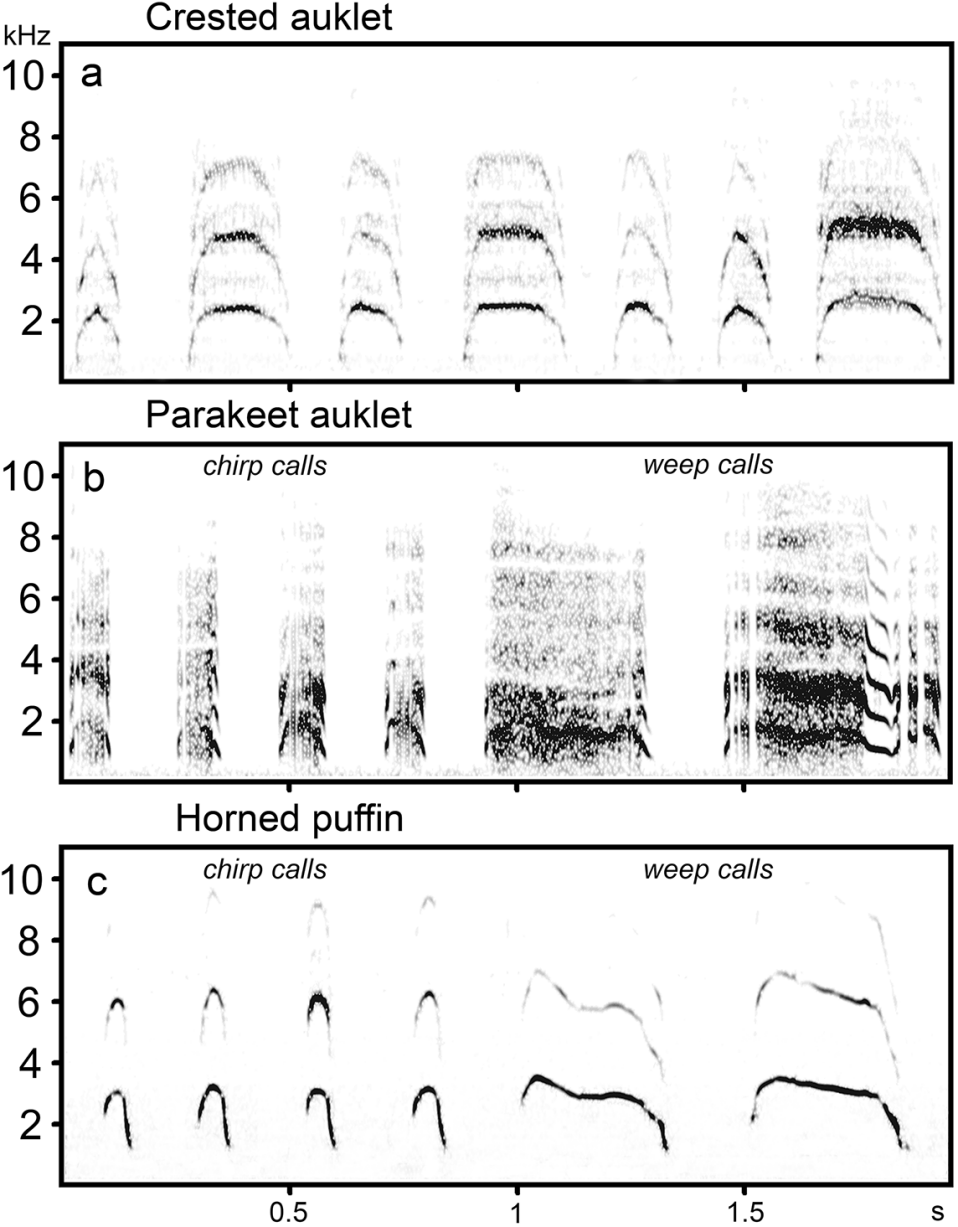
Spectrograms of calls in the vocal repertoires of auk chicks. (A) calls of crested auklet chicks, (B) chirp and weep calls of parakeet auklet chicks and (C) chirp and weep calls of horned puffin chicks. Spectrograms were created with a 1024-point FFT, Hamming window, frame 25% and 98.43% overlap.

According to visual inspection of spectrograms of chick calls, I recognized two main call types in the vocal repertoires of parakeet auklet and horned puffin chicks, and one highly variable call type in crested auklet chicks (Fig 1); [30]. Chirp calls of parakeet auklet and horned puffin chicks were brief, usually uttered rapidly and rhythmically in series, and their spectrograms had the appearance of an inverted U; weep calls were more than three times longer than chirp calls and were given at irregular intervals, sometimes interspersed with series of chirp calls. Weep calls of parakeet auklet and horned puffin chicks were modulated in frequency only slightly. In the vocal repertoire of crested auklet chicks, extremes of brief and long calls resembled chirp and weep calls (respectively) of the other species, but they simply defined the end points of a continuum, and could not be considered as two distinct call types (Fig 1); [30]. Chirp and weep calls of my study species are similar to brief “peep” and long “screech” calls reported for chicks of the Atlantic puffin (*Fratercula arctica*) [26] and brief “chirp” and long “peep” calls of chicks of the rhinoceros auklet (*Cerorhinca monocerata*) [9]. Both chirp and weep calls occurred in all recordings.

For measurement I chose calls randomly from whole recording sessions and then picked up from them calls of good quality. Totally I randomly selected for measurement 20 calls of good quality of each type: it was calls recorded with high call-to-noise ratio, not broken by wind, and not overlapped by parental calls. For crested auklets, I selected only brief calls, to facilitate comparison with the chirp calls of other species. In total, I analyzed 1629 calls: crested auklet, 280 brief chirp-like calls; parakeet auklet, 349 weep and 360 chirp calls; and horned puffin, 320 weep and 320 chirp calls. I measured nine variables on each call: maximum fundamental frequency (F_max); minimum fundamental frequency (F_min); peak frequency (F_peak), representing frequency at the maximum amplitude of a call; the lower (Q 25), medium (Q 50), and upper (Q 75) quartiles of the mean power spectrum of the call; total call duration (Dur); and duration from the end of the measured call to the beginning of the next call (Dur to next). The duration of inter-call interval (Dur to next) was measured regardless of call type followed the measured call. I also measured entropy of the mean power spectrum (Entropy), which reflects how chaotic a sound is (pure tones have low entropy, while broadband sounds have high entropy). To measure fundamental frequencies and durations I used the reticule cursor in the spectrogram window of Avisoft; energy variables were measured in power spectrum window of Avisoft.

Chick calls in the study species have a relatively simple frequency contour, but sometimes contain multiple non-linear phenomena [30], (Fig 1), namely biphonations, sidebands, or deterministic chaos, which occur when aperiodic oscillations replace or add to quasi-periodic ones during sound production [34–37]. The presence of non-linear phenomena in chick calls complicated the measurements of fundamental frequency values. For this reason, to reduce the masking effect of deterministic chaos, I measured maximum and minimum fundamental frequencies using the Avisoft SAS-Lab Pro line tool, which shows the maxima of the instantaneous spectrum (in my recordings the energy peak usually reflects the fundamental frequency and not the harmonics). For calls with two independent fundamental frequencies in spectrum I measured the darkest band.

I conducted statistical analyses with STATISTICA 8.0 (StatSoft, Inc., Tulsa, OK, USA). All tests were two-tailed; significance was set at p<0.05. Measurements on all acoustic variables were normally distributed for each chick (p>0.05, Kolmogorov–Smirnov test). To compare the effects of factors “species”, “individual” and “experiment” on the acoustic variables I used GLMM (with species and experiment as fixed factors and individual as a random factor). In one model I examined the separate effects of species, individual (nested within the species) and experiment, and conjoint (full cross) effect of species and experiment on the acoustic variables of chick calls.

## Results

Calls of food-deprived chicks differed from control chicks in most of all acoustic variables, for both chirp and weep calls (Tables 1 and 2). For chirp calls, GLMM showed significant effect of experiment and individual for all the nine variables, but effect of species–on eight of nine variables, excluding medium quartile (Q 50). Comparison of F-ratios showed that the effect of experiment on chirp call variables was often (for eight variables of nine) stronger than the effect of the individual and frequently (for six variables of the nine) stronger than the effect of the species (Table 1). For weep calls, available from parakeet auklets and horned puffins, the effect of experiment was again relatively high and comparable with those of the species and individual. GLMM showed significant effect of experiment on seven variables from nine measured, significant effect of species–also on seven variables, and significant effect of individual–on all nine measured variables. Comparison of F-ratios showed that the effect of experiment on weep call variables was stronger than the effects of species and individual for three of nine call variables (Table 1).

**Table 1:** Results of GLMM for separate effects of species, individual and experiment and conjoint effect of species and experiment on the acoustic variables of chick’ chirp and weep calls of three auk species.

		Species	Individuality (nested in species)	Experiment	Species and experiment
**Chirp calls**		**F_2,933_**	**F_21,933_**	**F_1,933_**	**F_2,933_**
	**F_min**	830.4**	28.9**	13.2**	73.4**
	**F_max**	714.8**	48.6**	818.7**	19.8**
	**F_peak**	63.4**	12.5**	123.5**	6.9**
	**Dur**	296.7**	47.9**	190.3**	63.9**
	**Dur to next**	57.1**	20.2**	254.9**	51.3**
	**Q25**	218.9**	62.3**	187.6**	17.0**
	**Q50**	0.3 ns	42.5**	255.7**	24.4**
	**Q75**	166.1**	28.6**	358.3**	65.7**
	**Entropy**	198.9**	27.3**	320.1**	17.6**
**Weep calls**		**F**_**1,650**_	**F**_**15,650**_	**F**_**1,650**_	**F**_**1,650**_
	**F_min**	1590.9**	45.4**	0.1 ns	9.4*
	**F_max**	309.5**	51.5**	194.5**	0.0 ns
	**F_peak**	0.3 ns	29.2**	102.8**	1.5 ns
	**Dur**	0.4 ns	42.6**	0.1 ns	1.0 ns
	**Dur to next**	33.4**	17.9**	276.5**	19.6**
	**Q25**	42.6**	49.2**	147.8**	0.4 ns
	**Q50**	219.8**	91.3**	124.3**	13.7**
	**Q75**	850.9**	73.4**	63.6**	8.9*
	**Entropy**	179.9**	24.6**	98.4**	0.0 ns

**–p<0.001

*–p<0.05, ns–p>0.05. The largest values for F-estimates were highlighted in bold for each acoustic variable. Individual was used as a random factor and was nested within the factor species.

**Table 2:** Descriptive statistics (mean±SD) for the acoustic variables of chirp and weep calls recorded during natural feedings (control) and after food deprivation (experiment). Means (±SD) calculated for 140–180 calls of each type taken from each species (20 calls per type per chick).

		Crested auklet (n = 7 chicks)		Parakeet auklet (n = 9 chicks)		Horned puffin (n = 8 chicks)	
		control	experiment	control	experiment	control	experiment
**Chirp calls**	**F_min (kHz)**	0.71±0.09	0.81±0.21	0.77±0.12	0.75±0.12	1.24±0.24	1.06±0.22
	**F_max (kHz)**	1.94±0.48	3.02±0.86	1.96±0.27	2.56±0.93	3.09±0.48	3.92±0.62
	**F_peak (kHz)**	1.76±0.66	2.65±1.16	1.78±0.93	2.17±1.2	2.42±0.54	2.97±0.95
	**Dur (ms)**	102±27	141±57	92±22	100±23	79±17	84±9
	**Dur to next (ms)**	458±389	156±65	262±213	144±70	209±80	152±27
	**Q25 (kHz)**	1.45±0.3	1.90±0.66	1.38±0.29	1.54±0.56	1.85±0.37	2.10±0.64
	**Q50 (kHz)**	2.13±0.59	2.84±0.65	2.38±0.49	2.60±0.8	2.29±0.38	2.73±0.65
	**Q75 (kHz)**	3.09±0.59	4.02±0.63	3.71±0.46	3.82±0.86	2.70±0.47	3.47±0.63
	**Entropy**	0.50±0.09	0.60±0.09	0.60±0.07	0.64±0.08	0.60±0.07	0.69±0.07
**Weep calls**	**F_min (kHz)**	-	-	0.76±0.13	0.72±0.12	1.25±0.29	1.30±0.36
	**F_max (kHz)**	-	-	2.01±0.29	2.59±1.16	2.73±0.62	3.31±0.81
	**F_peak (kHz)**	-	-	1.99±0.93	2.67±1.5	2.10±0.44	2.61±0.61
	**Dur (ms)**	-	-	664±189	674±282	683±209	674±194
	**Dur to next (ms)**	-	-	1622±1063	591±64	1090±893	489±372
	**Q25 (kHz)**	-	-	1.63±0.29	1.96±0.76	1.79±0.32	2.14±0.55
	**Q50 (kHz)**	-	-	2.79±0.97	3.08±1.04	2.13±0.38	2.66±0.52
	**Q75 (kHz)**	-	-	4.17±1.06	4.38±1.06	2.81±0.70	3.28±0.54
	**Entropy**	-	-	0.61±0.09	0.66±0.10	0.53±0.10	0.58±0.10

**Fig 2:**
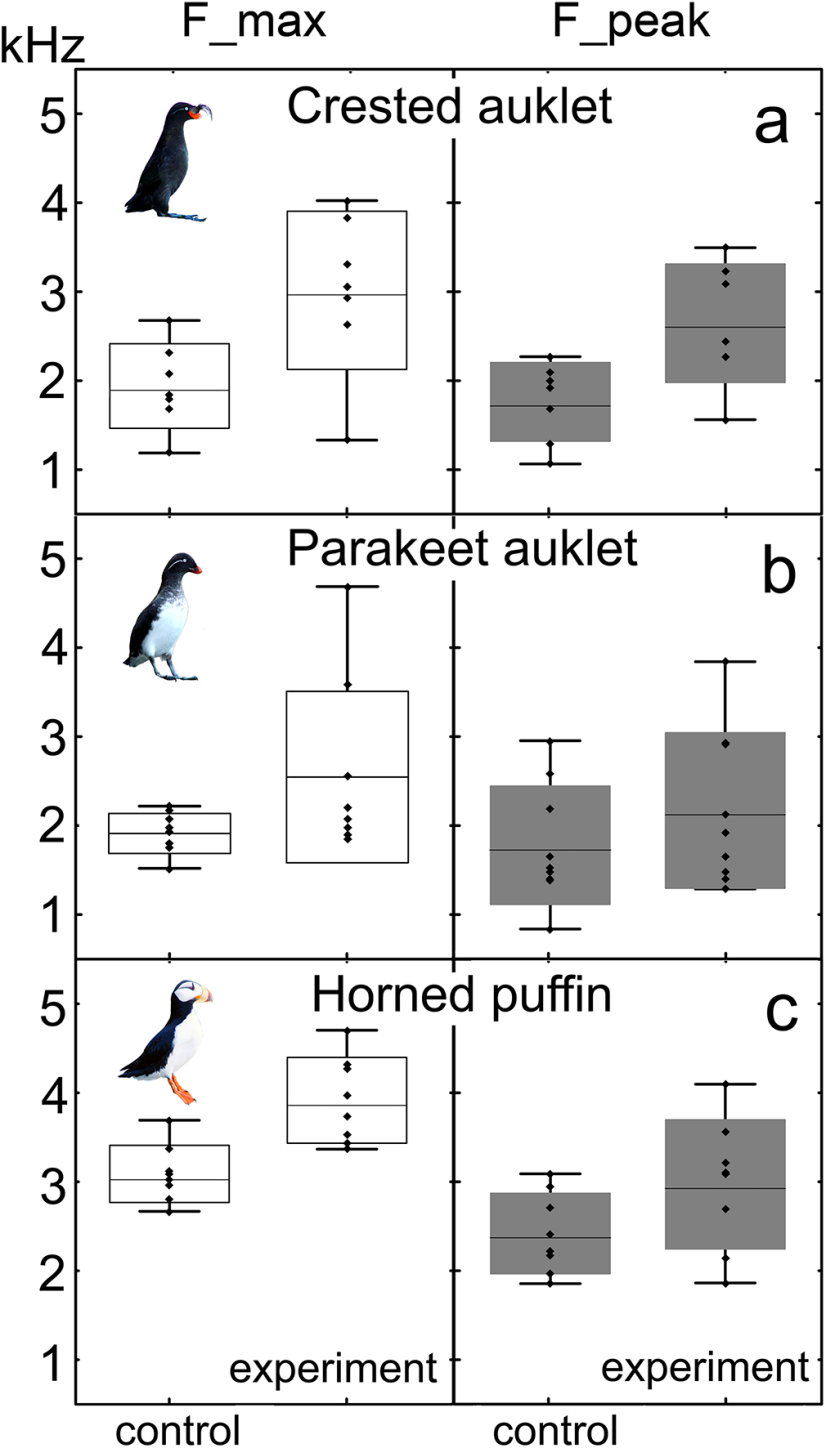
Shifts in maximum fundamental frequency (white boxes) and peak call frequency (dark boxes) across experimental conditions in chirp calls of crested auklet (A), parakeet auklet (B) and horned puffin (C). The boxes plotted for the average values of acoustic variables calculated for each chick. The middle points show the averages; box–SD; whiskers–minimum and maximum values; rhombs–the average variable value for each chick; all differences between control and experiment values of both frequency variables are significant (GLMM, p<0.05).

Overall, after food deprivation, hungry chicks of all three species emitted chirp and weep calls with higher F_max, F_peak, Q25, Q50, and Q75 (Table 2; S1 Table; Fig 2). After food deprivation total duration increased slightly but significantly in chirp calls but not in weep calls. At the same time, the interval between calls of food-deprived chicks was briefer than in control trials; i.e. the birds called at a higher rate (Table 2; S1 Table). Entropy increased slightly and significantly in both chirp and weep calls of all species, resulting in noisier and more broad-banded spectra (Table 2; S1 Table). F_min changed slightly and differently between control and experimental trials for chirp calls of all species, but not for weep calls (Table 2; S1 Table).

The variables that showed the greatest differences between control and experimental treatments were: F_max and Q 75 in chirp calls and F_max and Q25 in weep calls. The F_min and Dur were usually the least sensitive to the experimental conditions (Tables 1 and 2).

## Discussion

Almost all acoustic variables contained information about a chick’s hunger level in the three study auk species. Hungry chicks tended to utter calls more rapidly, and the calls were of higher fundamental frequency and with call energy shifted towards higher frequencies. In all study species, frequency variables changed more between control and food–deprived treatments than did calling rate or call duration.

My study extends findings on other species [2, 8–13], in showing that alcid chicks can change their call attributes and calling rate with increased nutritional needs. The results of the study show that food-deprived chicks called at a higher rate than control chicks. This is a widespread trend in both passerines [8, 10–14] and non-passerines [9, 16, 22, 25]. However, the increase in all main frequency variables and call entropy with increase of hunger has only been reported for a few species [13–15]. At the same time, no changes in frequency characteristics at all occur in other species [11; 16–19]. It is difficult to say why chicks of some species change only temporal organization of vocal begging (e.g. calling rate, call duration, number of calls), whereas chicks of other species also vary frequency characteristics. Petra Quillfeld with coauthors [19] supposed that the frequency variables may not be obligatory component of the signal of need in chicks of uniparous species. It is possible therefore that immediate assessment of chick condition through characteristics of single call elements is important mainly in the context of allocating food resources between siblings in multi-chick broods. Indeed, correlation between chick’ nutritional needs and call frequency variables were found in some passerines species with large broods [13, 14]. However, as in my study species, the Wilson’s storm-petrel (*Oceanites oceanicus*), raising only one chick, which communicates about hunger through changes in frequency attributes of begging calls [15] and our studies does not confirm the proposed hypothesis.

Another explanation of differentiation in vocal parameters used for need signaling consists with vocal individual recognition. Indeed, in many colonially breeding species, parents need to discriminate their mobile chicks from other conspecific young during pre-fledging and/or post-fledging periods [38–42]. In such cases chicks need to communicate vocally not only about its hunger, but also about its individuality. Frequency call parameters have been found to be an important cue for acoustic individual recognition between parents and young in many birds [39–41]. Few studies with special attention paid to comparison between indicators of need and individual identity in chick begging calls proved this hypothesis. Thus, in colonially breeding Jackson’s goldenbacked weaver (*Ploceus jacksoni*) some acoustic parameters of begging calls showed high individuality and were largely unaffected by a nestling’s state of hunger [43]. In thin-billed prion (*Pachyptila belcheri*) strong individual difference between chicks in main frequency characteristics have been also found, however these characteristics did not increase with hunger [19].

All my object auk species are diurnal and adults can find the way to the nest visually. So their chicks could use all the vocal variables for signaling of nutritional or other needs. Signaling about demands seems to be the main goal for the studied auk chicks, since the effect of hunger on vocal characteristics was almost always stronger than the effect of individuality and often stronger than the effect of species. The weak vocal individuality in chicks also occurs in some other colonial precocial species that have fixed nest sites. In these species parents can use topographical cues to find their chick, and this reduces selection for individualistic vocal traits [38–41]. Vocal individuality can be well expressed or considerably improved in a certain period of life when chicks are not attached to the fixed nest site or when parents keep feeding them after nest departure [38, 40, 42, 44–46]. Chicks of my study species do not leave their nest burrows until fledging, and the relationship between adults and juveniles apparently ceases immediately just after that event [20]. Therefore the highly individualistic calls of adults [47, 48] must appear later in development. However, the additional comparative studies of acoustic call variables from other bird species may give another useful clue on the evolution of cues to offspring fitness and demand in begging calls.

It should be noted however, that restricting access to the burrows in my experimental trials can affect not only the chick’s hunger level but also the motivational status of the parent. Perhaps parents may become less motivated to feed the chick when prevented from accessing the burrow due to, for example, stress or their own need to eat. So during the next feeding event parents could bring smaller portions of food or even visit the burrow without any food. And consequently chicks can react to these changes in parental behaviour with increase of their frequency call variables and calling rates. Future experiments with video recordings inside the nest burrows as those that were conducted with Atlantic puffin chicks [26] could help to prove or disprove this assumption.

Finally, my study confirms and extends a few other findings on alcids. In a study on the rhinoceros auklet, chick calling rate also increased with hunger level [9]. Atlantic puffin chicks emit fewer weep calls if they are well fed [26]. Those authors suggested that weep calls may signal hunger whereas chirp calls may signal chick quality [26]. In my study species, both chirp and weep calls were present in all recordings; in the rhinoceros auklet, weep calls are uttered mainly only early in life, and only chirp calls are given later [49]. A broader comparative survey of vocalizations and vocal development in the Alcidae in a phylogenetic context [50] and coupled with experiments with video recording during parental visits, would likely illuminate these agreements and inconsistencies.

## Supporting Information

S1 Table Descriptive statistics (mean±SD) for the acoustic variables of chirp and weep calls recorded during natural feedings (control) and after food deprivation (experiment). Means (±SD) calculated for 20 calls of each type taken from each chick.
(DOC)
