# Supplementary material for: Chick Begging Calls Reflect Degree of Hunger in Three Auk Species (Charadriiformes: Alcidae)
Source: PLoS One. 2015 Nov 4;10(11):e0140151. doi: 10.1371/journal.pone.0140151 (PMC4633236; doi:10.1371/journal.pone.0140151)
Supplement: S1 Table — Means (±SD) calculated for 20 calls of each type taken from each chick. (DOC) [file pone.0140151.s001.doc]

**S1 Table. Descriptive statistics (mean±SD) for the acoustic variables of chirp and weep calls recorded during natural feedings (control) and after food deprivation (experiment).** Means (±SD) calculated for 20 calls of each type taken from each chick.

|  |  |  | **Chick N** | **F_min (kHz)** | **F_max**  **(kHz)** | **F_peak**  **(kHz)** | **Dur**  **(ms)** | **Dur to next (ms)** | **Q25 (kHz)** | **Q50 (kHz)** | **Q75 (kHz)** | **Entropy** |
| --- | --- | --- | --- | --- | --- | --- | --- | --- | --- | --- | --- | --- |
| **Crested auklet** | **chirp calls** | **control** | 1 | 0.74±0.11 | 2.31±0.18 | 2.00±0.19 | 84±9 | 648±338 | 1.59±0.13 | 1.99±0.10 | 2.28±0.09 | 0.42±0.04 |
|  |  |  | 2 | 0.72±0.05 | 1.19±0.07 | 1.06±0.08 | 95±9 | 505±268 | 0.92±0.04 | 1.18±0.08 | 2.21±0.07 | 0.42±0.02 |
|  |  |  | 3 | 0.69±0.05 | 1.80±0.14 | 1.68±0.13 | 72±7 | 1051±547 | 1.53±0.12 | 1.82±0.10 | 3.16±0.11 | 0.46±0.06 |
|  |  |  | 4 | 0.66±0.06 | 1.85±0.17 | 2.27±0.74 | 93±18 | 188±58 | 1.63±0.09 | 2.74±0.10 | 3.53±0.05 | 0.60±0.02 |
|  |  |  | 5 | 0.82±0.08 | 2.68±0.25 | 1.29±0.47 | 108±11 | 248±85 | 1.10±0.05 | 2.03±0.13 | 3.35±0.21 | 0.64±0.02 |
|  |  |  | 6 | 0.66±0.07 | 2.08±0.35 | 2.10±0.74 | 146±32 | 319±98 | 1.71±0.18 | 2.13±0.43 | 3.38±0.34 | 0.45±0.07 |
|  |  |  | 7 | 0.68±0.06 | 1.69±0.13 | 1.93±0.75 | 113±12 | 246±45 | 1.63±0.09 | 3.01±0.08 | 3.73±0.27 | 0.54±0.04 |
|  |  | **experiment** | 1 | 0.83±0.15 | 3.05±0.25 | 2.45±0.47 | 112±25 | 230±51 | 1.87±0.40 | 2.72±0.16 | 4.40±0.54 | 0.61±0.06 |
|  |  |  | 2 | 0.64±0.04 | 1.33±0.10 | 2.27±1.03 | 78±18 | 207±63 | 1.15±0.06 | 1.90±0.12 | 3.30±0.38 | 0.50±0.03 |
|  |  |  | 3 | 1.16±0.16 | 2.93±0.20 | 1.56±1.71 | 103±22 | 170±72 | 1.65±0.33 | 3.06±0.51 | 4.08±0.40 | 0.64±0.04 |
|  |  |  | 4 | 0.82±0.11 | 2.63±0.19 | 3.22±1.15 | 121±56 | 107±38 | 2.34±0.22 | 3.53±0.62 | 4.86±0.51 | 0.64±0.06 |
|  |  |  | 5 | 0.86±0.12 | 3.83±0.17 | 3.09±0.99 | 162±21 | 130±33 | 1.43±0.13 | 2.49±0.23 | 3.58±0.18 | 0.62±0.05 |
|  |  |  | 6 | 0.79±0.12 | 4.02±0.23 | 3.50±0.41 | 230±21 | 123±24 | 3.08±0.31 | 3.55±0.19 | 3.97±0.21 | 0.47±0.05 |
|  |  |  | 7 | 0.55±0.10 | 3.31±0.34 | 2.44±0.66 | 183±15 | 121±45 | 1.76±0.36 | 2.65±0.32 | 3.96±0.51 | 0.68±0.03 |
| **Parakeet auklet** | **chirp calls** | **control** | 1 | 0.72±0.16 | 1.95±0.11 | 1.48±0.55 | 99±20 | 298±167 | 1.14±0.26 | 1.85±0.22 | 3.52±0.25 | 0.56±0.05 |
|  |  |  | 2 | 0.79±0.10 | 1.55±0.26 | 2.19±1.24 | 85±12 | 342±175 | 1.36±0.12 | 2.43±0.16 | 3.64±0.08 | 0.53±0.04 |
|  |  |  | 3 | 0.77±0.06 | 2.24±0.15 | 2.95±0.93 | 94±22 | 165±33 | 1.74±0.31 | 2.93±0.53 | 3.85±0.30 | 0.62±0.05 |
|  |  |  | 4 | 0.83±0.06 | 2.09±0.23 | 1.53±0.07 | 70±8 | 127±32 | 1.58±0.07 | 2.74±0.23 | 4.10±0.66 | 0.68±0.04 |
|  |  |  | 5 | 0.72±0.05 | 1.82±0.15 | 1.39±0.23 | 95±16 | 162±43 | 1.39±0.06 | 2.32±0.20 | 3.79±0.16 | 0.66±0.02 |
|  |  |  | 6 | 0.93±0.13 | 2.19±0.10 | 1.41±0.28 | 102±13 | 190±107 | 1.37±0.16 | 2.06±0.30 | 3.88±0.45 | 0.61±0.05 |
|  |  |  | 7 | 0.65±0.07 | 2.00±0.15 | 2.58±1.06 | 91±22 | 445±250 | 1.49±0.19 | 2.66±0.37 | 3.56±0.49 | 0.66±0.04 |
|  |  |  | 8 | 0.71±0.05 | 1.79±0.14 | 0.84±0.06 | 116±25 | 123±53 | 0.89±0.07 | 1.87±0.16 | 3.05±0.12 | 0.56±0.02 |
|  |  |  | 9 | 0.86±0.09 | 1.79±0.18 | 1.65±0.68 | 75±15 | 502±346 | 1.45±0.15 | 2.52±0.61 | 4.02±0.31 | 0.55±0.05 |
|  |  | **experiment** | 1 | 0.73±0.18 | 2.00±0.22 | 1.65±1.00 | 104±22 | 139±57 | 1.14±0.14 | 1.93±0.17 | 3.38±0.10 | 0.60±0.01 |
|  |  |  | 2 | 0.83±0.11 | 2.10±0.17 | 1.48±0.33 | 92±14 | 189±79 | 1.21±0.11 | 1.90±0.10 | 3.12±0.21 | 0.57±0.02 |
|  |  |  | 3 | 0.78±0.03 | 3.57±0.39 | 1.92±1.30 | 125±26 | 129±58 | 1.12±0.29 | 2.31±0.75 | 3.27±0.39 | 0.71±0.09 |
|  |  |  | 4 | 0.76±0.09 | 1.87±0.13 | 2.91±1.00 | 73±9 | 125±61 | 2.32±0.32 | 3.30±0.32 | 4.64±0.71 | 0.69±0.07 |
|  |  |  | 5 | 0.70±0.07 | 2.22±0.20 | 1.29±0.70 | 107±11 | 111±38 | 1.03±0.10 | 2.08±0.32 | 3.03±0.36 | 0.59±0.04 |
|  |  |  | 6 | 0.91±0.06 | 2.56±0.08 | 3.84±0.15 | 82±12 | 113±27 | 2.53±0.35 | 3.91±0.07 | 4.86±0.28 | 0.67±0.04 |
|  |  |  | 7 | 0.78±0.11 | 2.10±0.29 | 2.13±1.22 | 105±27 | 237±78 | 1.55±0.17 | 2.81±0.70 | 3.97±0.99 | 0.66±0.09 |
|  |  |  | 8 | 0.61±0.06 | 4.66±0.55 | 2.92±1.14 | 112±16 | 98±29 | 1.55±0.25 | 3.00±0.55 | 4.64±0.30 | 0.69±0.04 |
|  |  |  | 9 | 0.70±0.07 | 1.92±0.11 | 1.40±0.27 | 105±12 | 162±59 | 1.37±0.24 | 2.16±0.62 | 3.44±0.70 | 0.60±0.07 |
|  | **weep calls** | **control** | 1 | 0.67±0.12 | 2.30±0.13 | 1.93±0.13 | 466±72 | 2414±744 | 1.51±0.32 | 2.06±0.16 | 3.18±0.35 | 0.55±0.03 |
|  |  |  | 2 | 0.87±0.12 | 1.62±0.12 | 1.42±0.06 | 664±57 | 1835±448 | 1.46±0.04 | 2.33±0.22 | 3.53±0.14 | 0.46±0.06 |
|  |  |  | 3 | 0.83±0.12 | 2.18±0.11 | 1.94±0.11 | 820±187 | 795±431 | 1.62±0.21 | 2.28±0.21 | 3.84±0.21 | 0.62±0.03 |
|  |  |  | 4 | 0.84±0.07 | 2.18±0.13 | 1.72±0.25 | 603±89 | 774±661 | 1.42±0.09 | 2.08±0.14 | 4.44±1.11 | 0.65±0.05 |
|  |  |  | 5 | 0.61±0.06 | 2.01±0.24 | 3.12±1.82 | 699±135 | 787±322 | 1.91±0.24 | 3.66±0.66 | 5.17±0.79 | 0.75±0.07 |
|  |  |  | 6 | 0.88±0.06 | 2.20±0.05 | 1.92±0.06 | 778±65 | 1998±732 | 1.97±0.04 | 4.77±0.51 | 6.16±0.28 | 0.65±0.04 |
|  |  |  | 7 | 0.69±0.07 | 1.88±0.05 | 1.59±0.05 | 600±39 | 1929±715 | 1.47±0.10 | 2.15±0.29 | 3.37±0.27 | 0.60±0.04 |
|  |  |  | 8 | 0.72±0.08 | 2.14±0.27 | 2.76±1.22 | 938±113 | 1753±892 | 1.81±0.45 | 2.88±0.93 | 3.95±0.79 | 0.63±0.05 |
|  |  |  | 9 | 0.74±0.10 | 1.60±0.15 | 1.60±0.78 | 404±90 | 2046±1105 | 1.47±0.13 | 2.84±0.37 | 3.90±0.26 | 0.57±0.06 |
|  |  | **experiment** | 1 | 0.61±0.10 | 1.98±0.20 | 1.07±0.45 | 349±94 | 125±31 | 0.92±0.07 | 1.83±0.17 | 3.21±0.19 | 0.60±0.02 |
|  |  |  | 2 | 0.79±0.08 | 1.76±0.18 | 1.49±0.19 | 839±155 | 1796±638 | 1.44±0.11 | 2.15±0.18 | 3.16±0.12 | 0.49±0.05 |
|  |  |  | 3 | 0.85±0.11 | 2.44±0.06 | 2.55±0.93 | 940±220 | 483±257 | 1.96±0.20 | 2.65±0.57 | 3.95±0.26 | 0.76±0.07 |
|  |  |  | 4 | 0.65±0.05 | 1.55±0.17 | 2.52±1.08 | 1039±304 | 688±446 | 1.79±0.61 | 3.00±0.47 | 4.19±0.92 | 0.67±0.07 |
|  |  |  | 5 | 0.66±0.06 | 2.26±0.22 | 3.90±1.38 | 756±106 | 625±226 | 2.44±0.58 | 3.81±0.64 | 5.07±0.48 | 0.72±0.03 |
|  |  |  | 6 | 0.78±0.07 | 2.41±0.12 | 3.77±1.03 | 779±97 | 401±118 | 3.09±0.52 | 4.59±0.32 | 6.26±0.50 | 0.76±0.06 |
|  |  |  | 7 | 0.78±0.11 | 2.56±0.31 | 2.22±0.66 | 848±105 | 946±813 | 1.99±0.34 | 2.97±0.50 | 4.36±0.62 | 0.69±0.04 |
|  |  |  | 8 | 0.66±0.12 | 5.59±0.23 | 4.55±1.10 | 339±51 | 74±6 | 2.50±0.64 | 4.28±0.41 | 5.18±0.16 | 0.67±0.06 |
|  |  |  | 9 | 0.70±0.11 | 2.22±0.26 | 1.89±0.97 | 381±117 | 233±93 | 1.40±0.30 | 2.40±0.58 | 3.90±0.73 | 0.66±0.07 |
| **Horned puffin** | **chirp calls** | **control** | 1 | 1.44±0.07 | 2.96±0.54 | 1.96±0.29 | 75±5 | 264±75 | 1.61±0.08 | 2.00±0.11 | 2.33±0.08 | 0.58±0.05 |
|  |  |  | 2 | 1.13±0.18 | 3.08±0.30 | 2.70±0.18 | 80±14 | 283±105 | 1.83±0.22 | 2.40±0.19 | 2.82±0.17 | 0.66±0.06 |
|  |  |  | 3 | 0.94±0.14 | 2.67±0.26 | 1.86±0.17 | 89±11 | 137±24 | 1.50±0.12 | 1.86±0.08 | 2.26±0.15 | 0.62±0.04 |
|  |  |  | 4 | 1.16±0.15 | 3.12±0.31 | 2.22±0.35 | 64±8 | 221±42 | 1.63±0.11 | 2.10±0.08 | 2.56±0.32 | 0.63±0.04 |
|  |  |  | 5 | 1.18±0.13 | 3.69±0.53 | 3.09±0.28 | 76±10 | 174±34 | 2.64±0.15 | 3.02±0.15 | 3.44±0.31 | 0.61±0.07 |
|  |  |  | 6 | 1.07±0.04 | 2.81±0.26 | 2.18±0.02 | 107±20 | 165±20 | 1.68±0.12 | 2.12±0.04 | 2.30±0.04 | 0.51±0.04 |
|  |  |  | 7 | 1.38±0.18 | 3.37±0.44 | 2.94±0.55 | 76±6 | 162±21 | 2.00±0.16 | 2.52±0.19 | 3.08±0.40 | 0.64±0.05 |
|  |  |  | 8 | 1.60±0.05 | 3.02±0.29 | 2.41±0.53 | 64±8 | 264±96 | 1.90±0.13 | 2.31±0.29 | 2.79±0.35 | 0.59±0.06 |
|  |  | **experiment** | 1 | 1.09±0.07 | 3.37±0.58 | 1.86±0.31 | 88±10 | 177±14 | 1.52±0.30 | 2.01±0.18 | 2.67±0.44 | 0.68±0.04 |
|  |  |  | 2 | 1.42±0.07 | 3.43±0.29 | 2.70±0.91 | 85±10 | 212±23 | 1.74±0.09 | 2.36±0.18 | 3.26±0.34 | 0.72±0.05 |
|  |  |  | 3 | 1.05±0.09 | 3.54±0.26 | 3.10±0.68 | 83±10 | 149±15 | 1.32±0.19 | 2.04±0.14 | 3.01±0.49 | 0.69±0.05 |
|  |  |  | 4 | 0.82±0.10 | 3.73±0.40 | 2.15±0.63 | 80±6 | 149±24 | 1.76±0.10 | 2.27±0.15 | 3.29±0.49 | 0.67±0.07 |
|  |  |  | 5 | 0.90±0.18 | 4.32±0.61 | 3.56±0.49 | 88±7 | 119±17 | 2.77±0.42 | 3.45±0.33 | 4.02±0.35 | 0.69±0.09 |
|  |  |  | 6 | 0.93±0.15 | 4.27±0.54 | 3.21±0.86 | 86±12 | 145±25 | 2.22±0.35 | 2.93±0.24 | 3.67±0.54 | 0.71±0.06 |
|  |  |  | 7 | 1.05±0.13 | 3.98±0.38 | 3.09±0.75 | 88±9 | 132±17 | 2.41±0.45 | 3.06±0.33 | 3.58±0.20 | 0.63±0.10 |
|  |  |  | 8 | 1.22±0.18 | 4.70±0.23 | 4.10±0.57 | 79±7 | 131±19 | 3.03±0.28 | 3.69±0.22 | 4.29±0.22 | 0.69±0.06 |
|  | **weep calls** | **control** | 1 | 0.96±0.05 | 1.88±0.14 | 1.80±0.15 | 1018±157 | 2254±652 | 1.63±0.09 | 1.79±0.07 | 2.97±0.60 | 0.45±0.06 |
|  |  |  | 2 | 1.08±0.15 | 2.37±0.33 | 2.16±0.30 | 686±90 | 1299±603 | 1.74±0.26 | 2.11±0.24 | 2.41±0.29 | 0.53±0.08 |
|  |  |  | 3 | 1.03±0.17 | 2.40±0.55 | 1.95±0.21 | 803±149 | 1168±421 | 1.49±0.18 | 1.88±0.11 | 2.21±0.17 | 0.53±0.09 |
|  |  |  | 4 | 1.22±0.21 | 2.48±0.33 | 1.76±0.29 | 720±133 | 1986±1077 | 1.39±0.17 | 1.82±0.23 | 2.24±0.31 | 0.57±0.11 |
|  |  |  | 5 | 1.21±0.16 | 3.02±0.38 | 2.66±0.42 | 747±150 | 411±587 | 2.29±0.20 | 2.83±0.21 | 3.99±0.58 | 0.62±0.10 |
|  |  |  | 6 | 1.21±0.05 | 2.79±0.18 | 2.12±0.11 | 582±96 | 726±513 | 1.96±0.10 | 2.17±0.09 | 2.44±0.15 | 0.47±0.07 |
|  |  |  | 7 | 1.68±0.14 | 3.30±0.20 | 2.26±0.74 | 462±43 | 444±177 | 1.82±0.08 | 2.27±0.41 | 3.37±0.25 | 0.53±0.06 |
|  |  |  | 8 | 1.62±0.17 | 3.62±0.25 | 2.10±0.17 | 447±36 | 433±265 | 2.00±0.19 | 2.20±0.17 | 2.87±0.51 | 0.52±0.12 |
|  |  | **experiment** | 1 | 1.11±0.12 | 3.20±0.64 | 2.23±0.24 | 846±229 | 551±328 | 1.84±0.27 | 2.21±0.26 | 2.82±0.70 | 0.52±0.09 |
|  |  |  | 2 | 1.18±0.17 | 2.32±0.36 | 2.19±0.49 | 667±74 | 599±162 | 1.92±0.14 | 2.37±0.34 | 3.40±0.51 | 0.62±0.11 |
|  |  |  | 3 | 1.12±0.13 | 2.98±0.59 | 2.27±0.49 | 982±179 | 911±508 | 1.20±0.25 | 1.99±0.20 | 2.53±0.39 | 0.67±0.06 |
|  |  |  | 4 | 1.34±0.10 | 2.93±0.52 | 2.37±0.63 | 528±63 | 694±539 | 2.08±0.29 | 2.64±0.51 | 3.45±0.36 | 0.59±0.06 |
|  |  |  | 5 | 1.16±0.37 | 3.09±0.53 | 2.90±0.43 | 678±62 | 228±102 | 2.47±0.29 | 2.91±0.24 | 3.36±0.32 | 0.53±0.12 |
|  |  |  | 6 | 1.15±0.31 | 3.75±0.31 | 3.04±0.36 | 594±83 | 329±139 | 2.54±0.27 | 3.04±0.22 | 3.46±0.20 | 0.64±0.06 |
|  |  |  | 7 | 1.36±0.18 | 3.56±0.35 | 2.99±0.64 | 596±81 | 275±99 | 2.51±0.38 | 2.98±0.38 | 3.41±0.22 | 0.55±0.09 |
|  |  |  | 8 | 1.94±0.43 | 4.67±0.43 | 2.94±0.60 | 503±70 | 332±126 | 2.59±0.48 | 3.11±0.46 | 3.79±0.24 | 0.59±0.12 |
